# Rapid reconstitution of CD4 T cells and NK cells protects against CMV-reactivation after allogeneic stem cell transplantation

**DOI:** 10.1186/s12967-016-0988-4

**Published:** 2016-08-02

**Authors:** Julia Drylewicz, Ingrid M. M. Schellens, Rogier Gaiser, Nening M. Nanlohy, Esther D. Quakkelaar, Henny Otten, Suzanne van Dorp, Ronald Jacobi, Leonie Ran, Sanne Spijkers, Dan Koning, Rob Schuurman, Ellen Meijer, Floortje L. Pietersma, Jurgen Kuball, Debbie van Baarle

**Affiliations:** 1 Laboratory of Translational Immunology, Department of Immunology, Utrecht, The Netherlands; 2 Department of Haematology, Utrecht, The Netherlands; 3 Department of Virology, Utrecht, The Netherlands; 4 Department of Internal Medicine and Infectious Diseases, University Medical Center Utrecht, Utrecht, The Netherlands; 5 Theoretical Biology and Bioinformatics, Department of Biology, Utrecht University, Utrecht, The Netherlands; 6 Department of Haematology, VUMC, Amsterdam, The Netherlands; 7 Department of Immune Mechanisms, National Institute for Public Health and the environment (RIVM), Center for Infectious Disease Control, Antonie van leeuwenhoeklaan 9, Bilthoven, The Netherlands

**Keywords:** Stem cell transplantation, CMV, EBV, Immune reconstitution

## Abstract

**Background:**

Epstein-Barr virus and Cytomegalovirus reactivations frequently occur after allogeneic stem cell transplantation (SCT).

**Methods:**

Here we investigated the role of immune cell reconstitution in the onset and subsequent severity of EBV- and CMV-reactivation. To this end, 116 patients were prospectively sampled for absolute T cell (CD4 and CD8), B-cell (CD19) and NK-cell (CD16 and CD56) numbers weekly post-SCT during the first 3 months and thereafter monthly until 6 months post-SCT. Viral load was monitored in parallel.

**Results:**

In contrast to the general belief, we found that early T-cell reconstitution does not play a role in the onset of viral reactivation. CMV reactivation in the first 7 weeks after SCT however resulted in higher absolute CD8^+^ T-cell numbers 6 months post-SCT in patients with high-level reactivation, many of which were CMV-specific. Interestingly, rapid reconstitution of CD4^+^ T-cells, as well as NK cells and the presence of donor KIR3DL1, are associated with the absence of CMV-reactivation after SCT, suggestive of a protective role of these cells. In contrast, EBV-reactivations were not affected in any way by the level of immune reconstitution after SCT.

**Conclusion:**

In conclusion, these data suggest that CD4^+^ T-cells and NK cells, rather than CD8^+^ T-cells, are associated with protection against CMV-reactivation.

**Electronic supplementary material:**

The online version of this article (doi:10.1186/s12967-016-0988-4) contains supplementary material, which is available to authorized users.

## Background

Herpesvirus reactivations, both Epstein-Barr virus (EBV) [1] and Cytomegalovirus (CMV) [2, 3], occur frequently after allogeneic hematopoietic stem cell transplantation (SCT). Both viruses persist lifelong in the host in whom there is a tightly regulated balance between the virus infected cells and control by cytotoxic T-cell responses [1, 4, 5]. However, during the immunosuppressive state following SCT, viral reactivation can cause severe complications. Early diagnosis and treatment is considered crucial in order to prevent EBV-associated post-transplant lymphoproliferative disorders (EBV-PTLD) and CMV disease related mortality after SCT [6].

Frequent monitoring of EBV and CMV-DNA loads post transplantation is used to detect viral reactivations and infections providing a basis for pre-emptive therapy to prevent clinical complications [7, 8]. Both CD8^+^ T-cells as well as Natural Killer (NK) cells are key effector cells in eliminating virus-infected cells [9, 10]. For EBV it has been shown that incorporating T-cell reconstitution data increases the positive predictive value for development of EBV-PTLD [11]. Also for CMV, recovery of CMV-specific T-cells to levels greater than 10 × 10^6^/l were shown to be associated with protection from CMV-disease [12]. As measuring antigen-specific T-cell responses is often elaborate and patient-specific reagents are needed, a more general measure for immune reconstitution, including absolute cell counts, would be favored. In this light, Annels et al. have indicated an arbitrary value of sufficient T-cell reconstitution for which pre-emptive therapy can be withheld upon EBV-reactivation [13]. However, insufficient data exist on the relationship between viral reactivations and immune reconstitution. Even more, no comparative data exists on the role of different viruses on immune reconstitution in a single study.

Therefore, we here investigated the relationship between immune reconstitution and viral reactivation for both EBV and CMV separately in a prospective study in 116 allogeneic SCT recipients. We measured absolute CD4^+^ and CD8^+^ T-cells, CD16^+^ and CD56^+^ NK cells, and CD19^+^ B-cells weekly during the first 12 weeks post-SCT and thereafter at a monthly basis. We show that presence of donor inhibitory killer cell immunoglobulin-like receptor (KIR) KIR3DL1, rapid reconstitution of CD4^+^ T-cells and NK cells early after SCT is protective against CMV reactivation. In contrast, CMV reactivation early after SCT results in an enormous increase in total CD8^+^ T-cells 6 months post-SCT, many of which are CMV-specific. We did not observe any link between immune reconstitution dynamics and EBV reactivations in our cohort.

## Methods

### Study population and study design

116 patients receiving allogeneic SCT between January 2007 and June 2009 were prospectively followed during 6 months post-SCT at the department of Haematology of the University Medical Center Utrecht. Blood samples were drawn weekly from all patients to determine EBV and CMV load. After removal of plasma for EBV and CMV PCR analyses, the leftover whole blood was used in this study. The research was approved by the UMCU ethics committee. Written informed consent was obtained from all patients and studies were conducted in accordance with the guidelines of the World Medical Association’s Declaration of Helsinki.

### CMV and EBV-monitoring

CMV and EBV-monitoring was based on real-time TaqManTM CMV or EBV DNA PCR assay in ethylenediaminetetra acetic acid (EDTA)-plasma [14–16] performed weekly in all patients until 4 months post-transplantation. Patients were treated pre-emptively with valganciclovir (900 mg twice daily) when CMV-DNA load exceeded 500 copies/ml and with Rituximab 375 mg/m2 when EBV-DNA exceeded 1000 copies/ml. Valaciclovir prophylaxis was given to all patients (500 mg twice daily). Viral reactivations and/or infections were defined as EBV and or CMV viral load exceeding the detection limit of 50 copies/ml in plasma.

### Absolute immune cell counts

To determine the absolute number of immune cells per µl whole blood, TRUcount™ tubes (BD Biosciences (BD), San José, California, USA) were used according to manufacturers’ protocol. In brief, 50 µl of whole blood was incubated with CD45-PerCP (BD), CD3-Pacific Blue (eBioscience Inc., San Diego, California, USA), CD8-APC-Cy7 (BD), CD4-PE-Cy7, CD16-PE, CD19-FITC and CD56-APC. Thereafter erythrocytes were lysed (BD lysisbuffer) and samples were measured on LSR-II FACS machine. At least 2000 lymphocytes were measured (identified as CD45^+^SSC^low^) and analyzed with FACSdiva software (BD). We gated on CD3^+^CD4^+^ T-lymphocytes, CD3^+^CD8^+^ T-lymphocytes, CD3^−^CD16^+^ NK cells, CD3^−^CD56^+^ NK cells, and CD19^+^ B cells.

### Statistical analysis

All transplantation related risk factors for the development of CMV- or EBV-reactivation were assessed using a Fisher’s Exact test. We investigated the effects of CMV/EBV-reactivation on immune reconstitution using piecewise linear mixed models. For all subsets, two slopes were considered: one for early changes and one for the long-term trend. The time for the slope’s change (t = 7 weeks) was determined for all patients by a likelihood profile. The correlation between individual baseline values and the subsequent slopes was handled through the unstructured covariance matrix of random effects. Models were adjusted for age at SCT (<50 and ≥50 years), gender and ATG administration. The effects of immune reconstitution on CMV/EBV-reactivation were studied with Cox proportional-hazards models using time-dependent covariates (taking into account the marker changes over time). Cumulative incidence of viral reactivation were estimated by Kaplan–Meier analysis and were compared using the log-rank test.

P < 0.05 were considered statistically significant. All statistical analyses were conducted using SAS version 9.2 (SAS Institute, Cary, North Carolina, USA) and SPSS version 20.0.0 (SPSS Inc, Chicago, Illinois, USA).

## Results

### Patient population

**Fig. 1:**
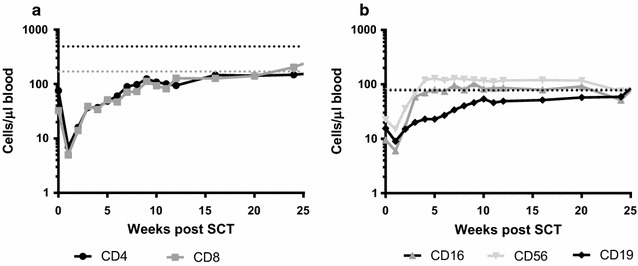
Reconstitution dynamics for the whole patient population. Absolute cell counts were determined weekly during the first 12 weeks and thereafter at a monthly basis. In (**a**) the median value for CD4^+^ and CD8^+^ T cells are plotted per time point. Lower normal values for healthy controls, based on Jentsch-Ullrich et al. (Clin Immunol 2005) and Comans-Bitter et al. (J Pediatr 1997), are depicted with a *dashed line*. Similarly, in (**b**) the median value for CD16^+^ and CD56^+^ NK cells, as well as CD19^+^ B cells are plotted per time point

116 patients receiving allogeneic stem cell transplantation between January 2007 and June 2009 were prospectively followed during the first 6 months post-SCT for EBV- and/or CMV-infections or reactivations. Patient and transplantation related characteristics are described in Table 1. Patients received an allogeneic SCT from either a related (n = 35) or an unrelated (n = 81) donor. The stem cell source was mostly peripheral blood (n = 106) and in most patients a nonmyeloablative conditioning regime was used (n = 106). In vivo T-cell depletion with anti-thymocyte globulin (ATG) was added to the conditioning regimen in patients receiving grafts from unrelated donors or HLA mismatched donors (n = 87). Transplantation associated risk factors that could influence the onset of viral reactivation were assessed in univariate analysis (Table 1). Only CMV serostatus of patient and donor (p < 0.0001) and ATG administration (p = 0.03) were significantly associated with onset of viral reactivation. Figure 1 shows the reconstitution dynamics after SCT of median CD4^+^ and CD8^+^ T-cells (Fig. 1a), B-cells and NK cells (Fig. 1b) for all patients. All subsets except CD4^+^ T-cells reached lower normal levels within 6 months post-SCT. NK cells reconstituted fastest, within 5 weeks after SCT their levels reached normal values and stabilized. CD4^+^ and CD8^+^ T-cells reconstituted similarly, though CD4 levels remained below normal. B-cells reconstituted more gradual and reached near normal levels at the end of follow-up.

**Table 1:** Patient characteristics

	Total	Reactivation				p value^a^
		Total (%)	CMV only	EBV only	CMV and EBV	
Number of patients	116	54 (47)	36	9	9	
Sex						
M	71	30 (42)	18	8	4	0.26
F	45	24 (53)	18	1	5	
Median age (range)	49.8 (17.6–70.6)	50.7 (17.6–68.5)	55.7 (28.5–68.5)	55.8 (17.5–68.3)	56.8 (23.8–65.8)	0.43
Stemcell source						
Cord blood	1	0 (0)	0	0	0	0.30
Peripheral blood	106	48 (45)	34	6	7	
Bone marrow	9	6 (67)	2	3	2	
Donor						
Related	35	13 (37)	11	1	2	0.23
Unrelated	81	40 (5)	25	8	7	
HLA mismatch	20	11 (55)	7	2	2	
Conditioning						
NMA	106	50 (47)	36	6	8	0.47
MA	10	4 (40)	0	2	2	
ATG	87	46 (53)	28	9	9	*0.03*
EBV serology (R/D)						
+/+	94	42 (45)	27	8	7	0.84
+/−	5	3 (60)	1	0	2	
−/+	4	2 (50)	2	0	0	
−/−	11	6 (54)	5	1	0	
CMV serology (R/D)						
+/+	39	28 (72)	24	0	4	*<10* ^*−4*^
+/−	37	18 (49)	10	5	3	
−/+	10	2 (20)	1	0	1	
−/−	28	5 (18)	1	4	0	
aGVHD						
Yes	64	29 (44)	17	5	7	0.83
No information	19	9 (47)	8	1	0	
Relapse	14	7 (50)	2	0	0	0.78
Viral load^b^						
Low (<1000 copies/ml)	NA	28 (52)	21	4	3	NA
High (>1000 copies/ml)	NA	26 (48)	15	5	6	

*ATG* anti-thymocyte globulin; *EBV* Epstein-Barr virus; *CMV* cytomegalovirus; *R/D* recipient/donor; *aGVHD* acute graft versus host disease; *NA* non-applicable

^a^Comparison between reactivation and no reactivation group: unpaired t test for age, univariate analysis using Fisher’s Exact test

^b^Patients were categorized in reactivation categories based on their peak viral load of either EBV and/or CMV DNA in plasma during 6 months post-SCT

### Viral reactivation

Patients were monitored during 6 months post-SCT for both EBV and CMV DNA in plasma. Viral infection or reactivation (viral load exceeding 50 copies/ml plasma) was diagnosed in 54 patients (47 %, Table 1). Twenty-eight patients (52 %) developed a low-level (load < 1000 copies/ml) viral reactivation and 26 (48 %, Table 1) a high-level (maximum load >1000 copies/ml). The median time to EBV-reactivation was 6 weeks (range 3–56) while it was 5 weeks for CMV reactivation (range 1–36 weeks). Among the CMV-reactivations, more than two-third (68 %) occurred during the first 7 weeks post-SCT. We observed two primary CMV infections who did not differ in term of time of viral infection and immune reconstitution from the rest of the patients.

### Increased CD8^+^ T-cell reconstitution after viral reactivation

**Fig. 2:**
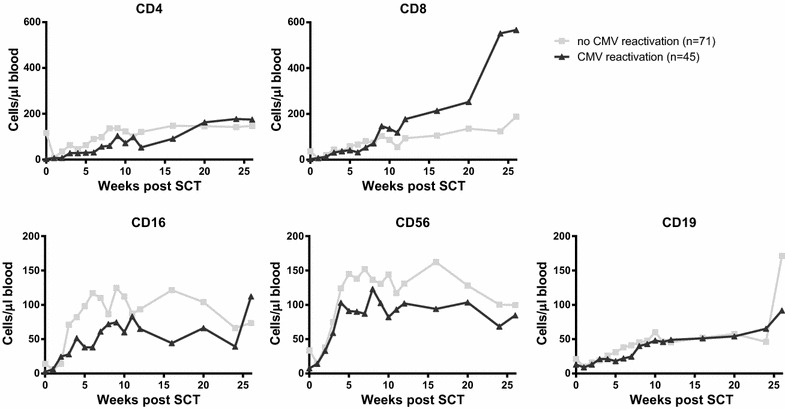
Longitudinal analysis of immune reconstitution dynamics for patients with or without CMV reactivation. Patients were subdivided based on whether or not they experienced CMV reactivation(s), based on CMV viral load exceeding the detection limit of 50 copies/ml in plasma. Data were analyses using piecewise linear mixed models with a two slope model. Reconstitution dynamics of CD4^+^ T cells, CD8^+^ T cells, CD16^+^ NK cells, CD56^+^ NK cells and CD19^+^ B cells are plotted per group. *Grey squares* depict the median value per time point for patients without CMV reactivation, *black triangles* depict the median value per time point for patients with CMV reactivation

**Fig. 3:**
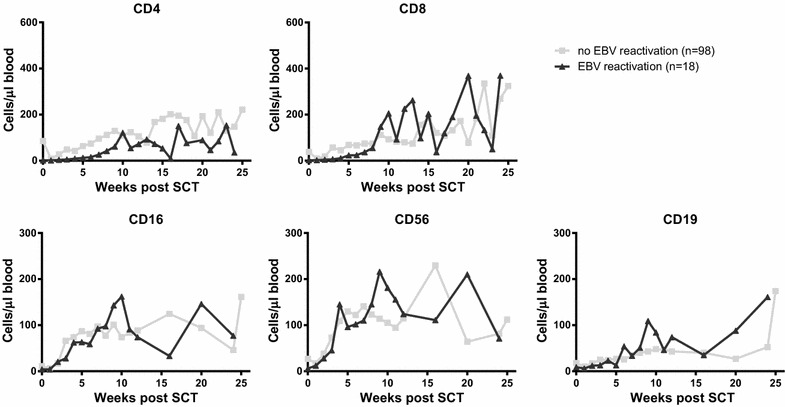
Longitudinal analysis of immune reconstitution dynamics for patients with or without EBV reactivation. Patients were subdivided based on whether or not they experienced EBV reactivation(s), based on EBV viral load exceeding the detection limit of 50 copies/ml in plasma. Data were analyses using piecewise linear mixed models with a two slope model. Reconstitution dynamics of CD4^+^ T cells, CD8^+^ T cells, CD16^+^ NK cells, CD56^+^ NK cells and CD19^+^ B cells are plotted per group. *Grey squares* depict the median value per time point for patients without EBV reactivation, *black triangles* depict the median value per time point for patients with EBV reactivation

To investigate whether the onset of viral reactivation after SCT influences the level of immune reconstitution, we used linear mixed effects models categorizing patients based on the occurrence of either EBV- or CMV-reactivation. No significant differences were observed in CD4^+^ T-cell and CD19^+^ B-cell reconstitution during the first 6 months post-SCT between patients with or without CMV-reactivation (Fig. 2). Remarkably, as reported recently by us for a retrospective cohort [17], the level of CD8^+^ T-cells 3–6 months post-SCT was significantly higher in patients with a CMV-reactivation compared to patients without in this larger prospective study (p < 0.0001). Moreover, patients with a CMV-reactivation showed a significantly lower level of NK cells (both CD16 and CD56) during the first months post-SCT compared to patients without CMV-reactivation (p = 0.007 and p = 0.018 respectively), which was independent of age, gender and ATG administration. Interestingly, when categorizing patients based on the occurrence of EBV instead of CMV-reactivation, no differences were observed in reconstitution dynamics for any of the cell types (Fig. 3).

Patients with CMV-reactivation showed significantly higher numbers of CD8^+^ T-cells at 6 months post-SCT (median 567, range 50–3589 CD8^+^ T-cells/µl) compared to patients without (median 188, range 12-713 CD8^+^ T-cells/µl; p < 0.0001). Our current prospective cohort with dense and extensive measurements allowed us to investigate if these high numbers were driven by the level and/or timing of CMV-reactivation. The highest numbers of CD8^+^ T-cells at 6 months post-SCT occurred in patients with a high-level CMV-reactivation (median 1419, range 295–3589 CD8^+^ T-cells/µl) (Additional file 1: Figure S1) and were threefold higher compared to healthy controls (average CD8^+^ T-cell number in healthy controls 395 cells/µl). Moreover, we found that patients with a CMV-reactivation during the first seven weeks post-SCT had higher CD8^+^ T-cell counts at 6 months post-SCT compared to patients with later CMV-reactivation (p < 0.0001). These data suggest that the observed increase in CD8^+^ T-cell numbers was the result of CMV-reactivation rather than playing a role in protection against CMV-reactivation. In contrast, EBV-reactivation seemed to play no role in CD8^+^ T-cell reconstitution.

### The level of CD4^+^ and CD16^+^ cells has prognostic value for the risk of CMV-reactivation

As we observed that NK cell levels during the first weeks post-SCT were higher in patients without CMV-reactivation, we used Cox proportional hazard models to investigate if the level of NK cells could be a predictor of the occurrence of subsequent CMV-reactivation. Indeed, with each increase of 50 CD16^+^ cells/μl, the risk of an early CMV-reactivation decreased with 20 % (HR: 0.800; 95 % CI [0.664; 0.963], Table 2). Interestingly, also a sufficient number of CD4^+^ T-cells was found to be associated with lower risk of CMV-reactivation: with each increase of 100 CD4^+^ T-cells/μl the risk of CMV-reactivation decreased with ~20 % (HR: 0.837; 95 % CI [0.704; 0.994], Table 2). No significant associations were found for the other subsets (Table 2).

**Table 2:** Cox proportional hazard analysis of the effect of reconstitution after SCT on the risk of CMV reactivation

	Increase of	Hazard ratio [95 % CI]	p value
CD4	100 cells	*0.837 [0.704;0.994]*	*0.04*
CD8	100 cells	0.982 [0.957;1.007]	0.16
CD16	50 cells	*0.800 [0.664;0.963]*	*0.02*
CD56	50 cells	0.877 [0.727;1.058]	0.17
CD19	25 cells	0.996 [0.984;1.009]	0.57

**Fig. 4:**
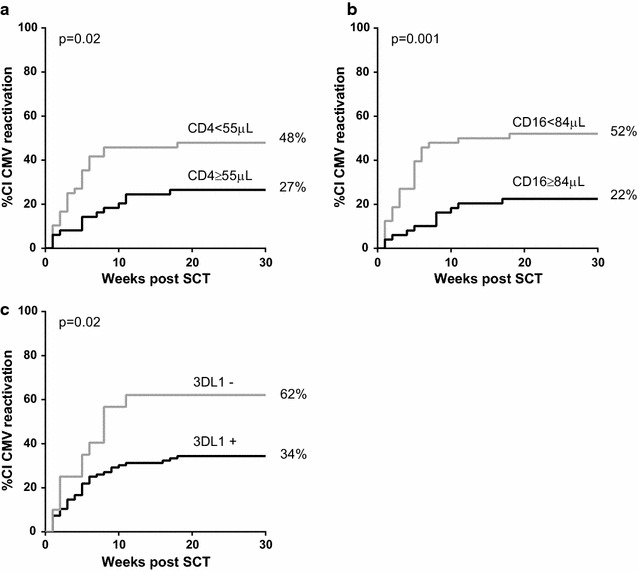
Cumulative incidence of CMV reactivation according to CD4^+^ and CD16^+^ reconstitution levels and presence of donor KIR 3DL1. **a** Patients were subdivided based on their CD4^+^ T cell count 5 weeks post-SCT. The cumulative incidence (CI) of CMV reactivation was plotted for patients with a CD4^+^ T cell count below or above the median value of 55 cells/μl. Similarly, in (**b**) patients were subdivided based on their CD16^+^ cell count 5 weeks post-SCT. The median value for the whole patient population was 84 cells/μl. **c** Patients were subdivided by the presence or not of donor inhibitory KIR 3DL1. CI of viral reactivation curves were estimated by Kaplan–Meier analysis and the comparison between the curves of the different groups was evaluated by the log-rank test

On average, CMV-reactivation occurred around 5 weeks post-SCT. Therefore, we next investigated whether CMV-reactivation occurs less frequently in patients with CD4^+^ T-cells and/or CD16^+^ NK cells above the median value observed at week 5 post-SCT. Indeed, the cumulative incidence (CI) of CMV-reactivation was significantly lower in patients with an absolute CD4^+^ T-cell count above 55 cells/μl (p = 0.019, Fig. 4a). Similarly, patients with a CD16^+^ cell count above 84 cells/μl had a significantly lower risk of CMV-reactivation (p = 0.0014; Fig. 4b).

Finally, we analyzed the impact of CMV-reactivation on the time to normalize cell counts. In line with our previous data we observed that patients with a CMV-reactivation tend to reach the lower bound of normal CD8^+^ T-cell counts faster compared with patients without a CMV-reactivation (p = 0.13, data not shown). On the other hand, patients with a CMV-reactivation tend to reach the lower bound of normal NK cell counts slower compared with patients without a CMV-reactivation (p = 0.13 and p = 0.05 for CD16^+^ and CD56^+^ respectively, data not shown). Even though a higher than median value of CD4^+^ T-cells 5 weeks post-SCT was associated with a lower risk of CMV-reactivation, almost none of the patients reached the lower bound of normal CD4^+^ T-cells during follow-up. Again, no difference was observed in time to normalize B-cells between patients with or without a CMV-reactivation.

### Genetic presence of donor inhibitory KIR 3DL1 is associated with a lower risk of CMV-reactivation

Both T and NK cells express killer cell immunoglobulin-like receptors (KIR) which bind to HLA molecules and can deliver inhibitory or activating signal to cells [18]. As patients at high risk of infection can be identified by genotyping KIRs, we next investigated if donor KIRs have a protective effect on CMV-reactivation. We found that patients with donor KIR of haplotype A (no other activating KIR than 2DS4) had a lower risk of CMV-reactivation than patients with donor KIR haplotype B (presence of activating KIR other than 2DS4) [HR: 0.427; 95 % CI (0.255; 0.885)]. We also found that the presence of the inhibitory KIR 3DL1 (n = 96) significantly reduced the risk of CMV-reactivation of ~80 % (HR: 0.243; 95 % CI [0.085; 0.691], Fig. 4c) while the KIR 3DS1 (n = 39), a common allelic variation of 3DL1, was not associated with CMV-reactivation. Moreover, the activating KIRs 2DS4 (n = 54), 2DS2 (n = 4) and their coexpression (n = 39) were not significantly associated with CMV-reactivation in our cohort.

## Discussion

Persistent viruses, like EBV and CMV, are normally controlled through cytotoxic T-cell responses [1, 4, 5]. Adequate T-cell reconstitution after SCT is therefore crucial in preventing viral reactivation progressing to severe complications [11, 12]. Here, we studied the role of immune reconstitution in the onset and severity of the viral reactivation. In contrast to the general belief, we found that early CD8^+^ T-cell reconstitution does not play a role in the early onset of viral reactivations. Proper reconstitution of CD4^+^ T-cells, as well as NK cells, and the presence of donor inhibitory KIR 3DL1, however significantly associated with a lower risk of CMV-reactivation after SCT.

CD4^+^ T-cell counts did not return to normal levels during follow-up, as also observed by others [19, 20]. In addition, we did not find a difference in CD4^+^ T-cell reconstitution between patients experiencing CMV-reactivation or not. We did however find a clear association between CD4^+^ T-cell reconstitution levels early after SCT and the risk of CMV-reactivation. The importance of proper CD4^+^ T-cell reconstitution and improved outcome after transplantation has been recently emphasized in a pediatric cohort, although this study did not investigate the role of CMV [21]. We also found that CD4^+^ T-cells tend to reconstitute better after CMV-reactivation in patients with early reactivation (not shown). These results suggest that patients with proper CD4^+^ T-cell reconstitution will have a lower risk of CMV-reactivation. However, if they do reactivate CMV, their CD4^+^ T-cell counts will increase and reach similar levels compared to patients without CMV-reactivation. In line with this, Berger et al. also showed that patients with low CD4^+^ T-cell count on day 35 post-SCT had a higher risk of dying of infections [19]. Even though our study was not designed to determine an actual cutoff value for CD4^+^ T-cells, nor for NK cells, to be protective against CMV-reactivation, we show that a patient with e.g. 100 NK cells/μl has a 20 % lower risk to reactivate CMV compared with a patient with only 50 NK cells/μl at that same moment.

The presence of donor activating KIRs has been described to protect against CMV-reactivation in solid organ and hematopoietic cell transplantation [22–25]. In our study, however, we did not find a protective effect of any donor activating KIRs (including KIR 2DS4 and 2DS2). It is described that the protective effect of KIRs might depend on their gene expression levels. Elevated KIR gene expression has been shown to be associated with risk of CMV-reactivation. These elevated activating KIR expressions in CMV-viremic SCT patients might be due to factors that activate CMV or are even initiated by CMV [26]. Interestingly, we found that donor KIR 3DL1 was associated with a lower risk of CMV-reactivation supporting a recent study that showed that in vitro 3DL1 expression correlated with lysis of CMV-infected fibroblasts [27]. Moreover, we found that patients with donor KIR haplotype A had a lower risk of CMV-reactivation than patients with donor KIR haplotype B. Although this result is in contradiction with published studies [23, 28], a recent study in renal transplant patients also showed that depending on HLA-type, KIR haplotype A might be protective against infection such as CMV [24].

It is well known that CMV-specific CD8^+^ T-cells are important in the control of CMV-reactivations after SCT. However, NK cells were shown to effectively control CMV infection even in the absence of T-cells [10]. We have previously reported the importance of gdT-cells after SCT to help resolving viral reactivation [17] (measured in a subgroup of the present study population) and it is therefore tempting to speculate that NK cells, together with CD4^+^ T-cells, are important for the initial control of CMV shortly after SCT, whereas CD8^+^ T-cells as well as gdT-cells, are most likely more important for the resolution of as well as toxicities due to viral reactivations. Several studies have indeed described the association between virus-specific T-cells and CMV or EBV-related complications [11–13, 29].

In our study we show a strong impact of CMV-reactivation on the number of total CD8^+^ T-cells following reactivation (at 6 months post-SCT). This could result in a false positive perception of adequate T-cell reconstitution. Although high numbers of T-cells are present, these are more often of an effector rather than a memory phenotype and contain many CMV-specific T-cells (data not shown and [30]). This may result in a restricted repertoire of the T-cell pool, which may be detrimental for the host. Indeed, Suessmuth et al. recently showed that CMV-reactivation results in a clonal expansion of CMV-specific T-cells and significant defects in the total CD8 Tem TCR repertoire [30]. Surprisingly, there were marked differences in reconstitution dynamics between patients with EBV- versus CMV-reactivation. The rapid expansion of T-cells seen in patients after CMV-reactivation was not observed in patients with high-level EBV-reactivations, suggesting that this rapid expansion is not a phenomenon caused by viruses in general but is CMV-specific.

In-vivo T-cell depletion through alemtuzumab has been shown to delay both CD4^+^ and CD8^+^ T-cell reconstitution and ATG administration results in a delayed CD4^+^ T-cell reconstitution [31]. Also in our study, we observed differences in T-cell counts early after SCT between patients who received ATG and patients that did not. Treatment with ATG resulted in delayed CD4 reconstitution, but increased CD8 T-cell numbers, irrespective of viral reactivation (data not shown). Although age is also known to have an impact on immune reconstitution [32–34], the associations between reconstitution dynamics and viral reactivation we observed here were also independent of recipients’ age.

## Conclusions

Based on these findings, we hypothesize that onset of CMV-reactivation is not influenced by a lack of CD8^+^ T-cell control, but rather by lack of sufficient numbers of CD4^+^ T-cells as well as NK cells early after SCT. CD8^+^ T-cells, on the other hand, significantly increase later after CMV-reactivation when most viral reactivations had resolved, and thus most likely play an important role in determining the outcome of reactivation in the long run. Moreover, the marked differences between EBV- and CMV-reactivation observed in this study calls for future studies investigating potential associations between other common viruses and immune reconstitution dynamics after SCT.

## Additional file

10.1186/s12967-016-0988-4 CD8+ T cell counts at 6 months post-SCT in patients with and without viral reactivation. Number of CD8+ T cell counts 6 months post-SCT in patients without viral reactivation (n = 33), with a low level of viral reactivation (viral load <1000 copies/ml, n = 12) and with a high level of viral reactivation (viral load >1000 copies/ml, n = 9).

## Abbreviations

EBV: Epstein-Barr virus
CMV: Cytomegalovirus
SCT: stem cell transplantation
EBV-PTLD: EBV-associated post-transplant lymphoproliferative disorders
NK cells: natural killer cells
KIR: killer cell immunoglobulin-like receptor
ATG: anti-thymocyte globulin
