# Supplementary material for: Rapid reconstitution of CD4 T cells and NK cells protects against CMV-reactivation after allogeneic stem cell transplantation
Source: J Transl Med. 2016 Aug 2;14:230. doi: 10.1186/s12967-016-0988-4 (PMC4971638; doi:10.1186/s12967-016-0988-4)
Supplement: Supplementary file 1 — 10.1186/s12967-016-0988-4 CD8+ T cell counts at 6 months post-SCT in patients with and without viral reactivation. Number of CD8+ T cell counts 6 months post-SCT in patients without viral reactivation (n = 33), with a low level of viral reactivation (viral load <1000 copies/ml, n = 12) and with a high level of viral reactivation (viral load >1000 copies/ml, n = 9). [file 12967_2016_988_MOESM1_ESM.pdf]

Supplemental Material

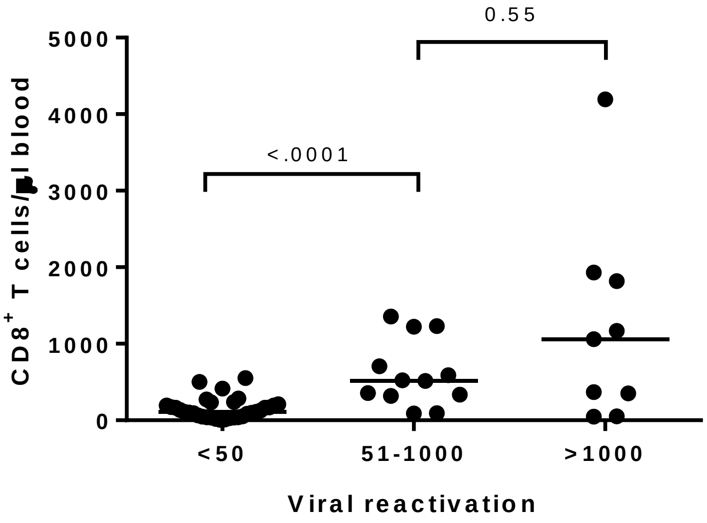

Figure S1. CD8$^+$ T cell counts at 6 months post-SCT in patients with and without viral reactivation. Number of CD8$^+$ T cell counts 6 months post-SCT in patients without viral reactivation (n=33), with a low level of viral reactivation (viral load<1000 copies/ml, n=12) and with a high level of viral reactivation (viral load>1000 copies/ml, n=9).
